# JunD/AP1 regulatory network analysis during macrophage activation in a rat model of crescentic glomerulonephritis

**DOI:** 10.1186/1752-0509-7-93

**Published:** 2013-09-22

**Authors:** Prashant K Srivastava, Richard P Hull, Jacques Behmoaras, Enrico Petretto, Timothy J Aitman

**Affiliations:** 1 MRC Clinical Sciences Centre, Imperial College London, Hammersmith Hospital, Du Cane Road, London, W12 0NN, UK; 2 Centre of Complement and Inflammation Research, Imperial College London, Du Cane Road, London, W12 0NN, UK

## Abstract

**Background:**

Function and efficiency of a transcription factor (TF) are often modulated by interactions with other proteins or TFs to achieve finely tuned regulation of target genes. However, complex TF interactions are often not taken into account to identify functionally active TF-targets and characterize their regulatory network. Here, we have developed a computational framework for integrated analysis of genome-wide ChIP-seq and gene expression data to identify the functional interacting partners of a TF and characterize the TF-driven regulatory network. We have applied this methodology in a rat model of macrophage dependent crescentic glomerulonephritis (Crgn) where we have previously identified JunD as a TF gene responsible for enhanced macrophage activation associated with susceptibility to Crgn in the Wistar-Kyoto (WKY) strain.

**Results:**

To evaluate the regulatory effects of JunD on its target genes, we analysed data from two rat strains (WKY and WKY.L*Crgn2*) that show 20-fold difference in their JunD expression in macrophages. We identified 36 TFs interacting with JunD/Jun and JunD/ATF complexes (i.e., AP1 complex), which resulted in strain-dependent gene expression regulation of 1,274 target genes in macrophages. After lipopolysaccharide (LPS) stimulation we found that 2.4 fold more JunD/ATF-target genes were up-regulated as compared with JunD/Jun-target genes. The enriched 314 genes up-regulated by AP1 complex during LPS stimulation were most significantly enriched for immune response (P = 6.9 × 10^-4^) and antigen processing and presentation functions (P = 2.4 × 10^-5^), suggesting a role for these genes in macrophage LPS-stimulated activation driven by JunD interaction with Jun/ATF.

**Conclusions:**

In summary, our integrated analyses revealed a large network of TFs interacting with JunD and their regulated targets. Our data also suggest a previously unappreciated contribution of the ATF complex to JunD-mediated mechanisms of macrophage activation in a rat model of crescentic glomerulonephritis.

## Background

Recent development of high throughput profiling methods such as microarray, RNA-seq and chromatin immuno-precipitation followed by microarray (ChIP-chip) or sequencing (ChIP-Seq) have revolutionized the study of protein–DNA interactions and have made information regarding gene expression and transcription factor binding sites (TFBS) readily available. Positional binding of transcription factors (TF) can vary from the basal promoter region to a few kilobase pairs from the transcription start site (TSS). When a TF is bound to the promoter region, experimental evidence suggests an increased probability of the gene being its target, but the functional nature of the TFBS would be difficult to characterise based upon the TFBS profile alone. Therefore, the task of associating TFBS with a gene is not trivial. Traditionally, identified TFBS have been associated with the nearest gene [1]. However, the effect of functional TFBS can be observed in a target’s gene expression, and therefore integration of ChIP-Seq profiles with gene expression is crucial for identification of active binding sites. There have been several previous attempts at dataset integration, some of which have tried to apply regression based models [2], while some have validated the ChIP-Seq identified targets by transcriptionally silencing the TF and observing the effect on gene expression [3,4]. Whilst most of the methodology developed for integrating the two datasets considers genomic attributes from the ChIP-seq dataset, none of the methods are based on the TF’s interacting partners or interactome.

The function and efficiency of a TF is often modulated by its interaction with other proteins to achieve finely tuned regulation of gene expression. TF complexes can be inferred by assessing significant enrichment of motif spacing within ChIP-Seq peaks [5]. Such TF complexes are often referred to as *cis-*regulatory modules. Several studies have tried to develop computational frameworks for the identification of relevant TF complexes for co-expressed genes [6,7], and it has been previously established that if genes are under a common regulatory mechanism they tend to follow similar expression patterns. In this study, we have developed a computational framework which enables integration of the TF-complex obtained from a ChIP-Seq genome-wide TF binding profile with gene expression profiles.

We have devised and tested the new framework using a rat model for crescentic glomerulonephritis (Crgn). Crgn is an important cause of kidney failure, for which the underlying molecular basis is largely unknown. The Wistar-Kyoto (WKY) rat is uniquely susceptible to experimentally induced Crgn [8]. We previously investigated the genetic basis to Crgn susceptibility in crosses between the Crgn-sensitive WKY and the Crgn-resistant Lewis (LEW) rat strains. Crgn was linked to 7 quantitative trait loci (QTLs) including the chromosome 16 QTL *Crgn2*, in which the AP-1 transcription factor JunD was identified as a primary determinant of macrophage activation and associated with Crgn susceptibility [9]. WKY bone marrow-derived macrophages (BMDMs) demonstrated marked overexpression of JunD and increased Fc receptor-mediated macrophage activation compared with BMDMs from LEW and from congenic rats (WKY.L*Crgn2*) in which the LEW *Crgn2* QTL was introgressed onto the WKY background. Therefore, characterisation of JunD’s physical and genetic interactions may provide key insights into complex biological systems. In our previous study, we have shown that JunD is a regulator of oxidative stress and IL1 beta synthesis in macrophages [4].

In this study, by characterising the JunD interactome and modelling gene expression data, we have identified a group of genes that show differential co-expression and enrichment during LPS stimulation between WKY and the WKY.L*Crgn2* congenic strain. Our data also suggests that interaction of JunD with specific TFs could be important for the over-activation phenomenon of macrophages in the WKY strain. This work suggests that the JunD complex has modulatory effects on macrophage gene expression which provides the basis for understanding *JunD-*mediated macrophage activation, and enabling identification of novel targets for modulating macrophage function.

## Methods

### ChIP-Seq and gene expression data

Bone-marrow derived macrophages (BMDM) were isolated from WKY and WKY.L*Crgn2*. BMDMs were subjected to LPS stimulation (additional details on the efficiency of the LPS treatment can be found in Hull et al. [4]). Gene expression profiles were generated using Rat Gene 1.0 ST arrays (Affymetrix, Santa Clara, CA, USA) at 0, 2, 4 and 8 hours after LPS stimulation using 4 biological replicates for each time point. Sample pre-processing and hybridisation was performed as per manufacturer’s recommendations. The Affymetrix .CEL files were imported to R statistical software version 2.11, using R Affy package version 1.34. Probe annotation was done using custom chip definition files [10], and probesets with single nucleotide polymorphisms between Brown Norway (reference genome) rat strains and WKY rat strains were removed. Background correction was performed using robust multichip average (RMA) [11] and data was normalised using the quantile normalisation method. Differential expression analysis was performed using the Bioconductor package SAMR, which implements SAM (Significance analysis of microarray) statistics in R [12], and a cut-off of 5% false discovery rate (FDR) was applied.

Genome-wide JunD TF binding profiles were generated for WKY and WKY.L*Crgn2* at two initial time-points. ChIP was performed with a JunD antibody (Santa Cruz sc74-X) and a negative IgG control (sc-2026). ChIP-Seq peaks were predicted using BayesPeak version 1.13 [13], and predicted peaks with a posterior probability greater than 0.9 were considered significant (for experimental details please refer to Hull et al. [4]).

### Motif analysis

De novo and known TFBSs were identified using the HOMER software package version 2 [14]. De novo motif analysis was performed using default parameters expecting a 12 bp motif. TFBS were identified using the area underneath the ChIP-Seq peaks using the publically available software Transfac transcription factor matrices version 6 [15].

### Inferences of TF targets using gene expression data

TF complexes were inferred using the methodology described in [16]. The primary focus of Banerjee et al. was TFBS in promoter regions. In this study we have extended this methodology to the area under predicted ChIP-Seq peaks. A TF pair was considered to be co-operativly interacting if the expression correlation scores of genes showing binding of both TFs were significantly greater than any set of genes with binding of either TF alone. We used the proposed model based on the multivariate hypergeometric distribution.

### TF targets using ChIP-Seq data

Spaced motif analysis was carried out using the SpaMo software [5]. In brief, the SpaMo algorithm predicts transcription factor interactions by assessing significant enrichment of motif spacings within the ChIP-Seq peaks. Primary and secondary motifs for the motif spacing analysis were retrieved from the public Transfac database [15]. Here we have used a window of 50 bp on either side of the primary motif to look for significant enrichment of motif spacings with a secondary motif. All reported P-values were adjusted for the number of intervals and motifs tested using the Bonferroni correction.

### Integration of gene expression with ChIP-Seq profiles

AP-1 complex TF gene expression profiles were modelled based on the Gaussian process differential equations [17]. This methodology was specifically developed to model short time-series datasets and is implemented in tigre, R package [17]. This was used for ranking the expressed set of transcripts on the microarray based on the similarity of the gene expression profiles represented in the form of likelihood scores. The ranked sets of transcripts were subjected to gene set enrichment analysis using likelihood scores based on a pre-ranked list and SpaMo identified TF-pair target genes as datasets [18,19]. A dataset was considered significant if it had a family wise error rate (FWER) < 0.05.

## Results

### Identification of JunD-enriched sites by ChIP-Seq and characterisation of targets

**Figure 1:**
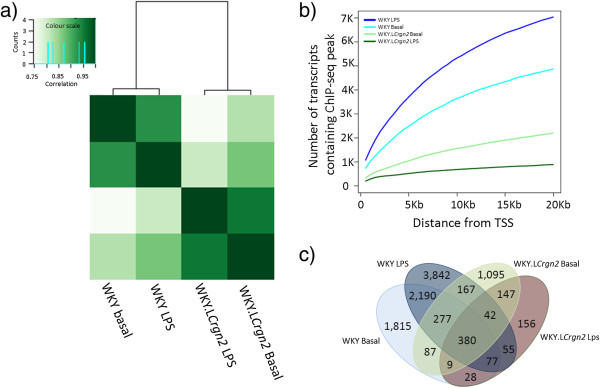
**Comparison of ChIP-Seq experiments under different conditions. a)** Clustering of ChIP-Seq data based upon correlation coefficient calculated for the number of reads under the predicted peaks. JunD ChIP-Seq profiles are able to distinguish the WKY from WKY.L*Crgn2*. **b)** Illustration of the relationship between the number of transcripts containing at least one ChIP-Seq peak and its distance from the TSS (in Kb). Peaks were linked to a gene if they were located within ± 20Kb of the transcription start site (TSS). The lines coloured in cyan, blue, light green and green represent ChIP-Seq profiles for WKY basal, WKY LPS, WKY.L*Crgn2* basal and WKY.L*Crgn2* LPS respectively. The number of transcripts increases in WKY after LPS stimulation while in the case of WKY.L*Crgn2* it decreases. This suggests an impaired role of JunD/AP-1 complex during LPS stimulated macrophage activation. **c)** Venn diagram showing the overlaps between different conditions. The numbers represent the transcripts associated with peaks.

Genome-wide chromatin occupancy by JunD was determined in WKY and WKY.L*Crgn2* BMDMs for the basal resting state and after 2 hrs of LPS stimulation. The ChIP-Seq peaks were identified using BayesPeak [13] with a significance threshold of 90% posterior probability. For BMDMs with the higher levels of *JunD* expression in the WKY strain, we identified 27,126 (basal) and 36,688 (LPS stimulated) peaks, while for the WKY.L*Crgn2*, 16,594 and 8,690 peaks were detected for the basal and LPS stimulated datasets respectively. *De novo* motif analysis as well as investigation of known JunD TFBS showed significant enrichment of JunD motifs in all 4 datasets *De novo* motif analysis showed an enrichment P-values of 3.1e-87, 1.4e-44 and 2.6e-34 for WKY basal, WKY LPS and WKY.L*Crgn2* basal conditions respectively. While enrichment analysis for the known AP1 motif showed an enrichment P-values of 4.5e-13, 5.3e-23, 3.0e-6 and 6e-13 for WKY basal, LPS and WKY.L*Crgn2* basal, LPS stimulated conditions respectively [4]. Reads that mapped to ChIP-Seq peaks, irrespective of condition or strain were clustered using correlation, showing that the ChIP-Seq profiles obtained were able to distinguish WKY from WKY.L*Crgn2* irrespective of LPS stimulation (Figure 1a). Within clusters the correlation was >0.95 while across the clusters correlation was considerably lower (<0.75). Identified peaks were mapped to the nearest gene’s TSS within a window spanning ±20 kb. This resulted in the identification of 4,864, 7,031, 2,205 and 895 transcripts for WKY basal, LPS, WKY.L*Crgn2* basal and LPS datasets respectively (Figure 1b and c). The transcript associated peaks were validated in triplicate an independent set of rats and we were able to validate 78% of the randomly selected peaks [4]. The number of transcripts associated with the peaks increases after LPS stimulation in the case of WKY while it decreases in the case of WKY.L*Crgn2*, suggesting a diminished role of JunD-driven activity in WKY.L*Crgn2* during LPS stimulation. This observation is also consistent with the increase in *JunD* expression levels following LPS stimulation as shown previously and reported in [4].

### Inferring TF interactions with JunD using gene expression data

**Figure 2:**
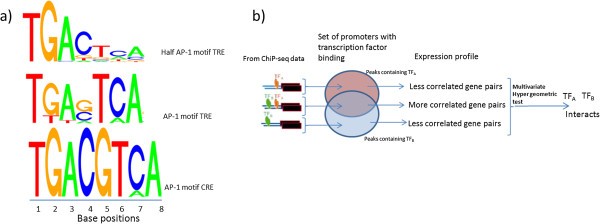
**JunD preferred TFBS motifs and its interaction with other TFs. a)** JunD primarily recognises two types of motifs: (TPA)-responsive element (TRE; this element has the base sequence TGA[C/G]TCA) and cAMP-responsive element (CRE; this element has the base sequence TGACGTCA). Literature also suggests that JunD is capable of binding conserved TGAC and less conserved TCA motifs as well, and this is often referred to as a half AP-1 binding site. CRE motifs are preferred when JunD forms a heterodimer with the ATF family of transcription factors. **b)** TFs often interact with other TFs or proteins. It has been shown before that the if two TFs, say TF_A_ and TF_B,_ co-operatively interact with each other than the target gene pairs for the individual TFs are less correlated compared to the gene pairs that contain both TFBS sites. Here we have employed a similar methodology for sequences under the peak to characterise their interactions. We have employed a previously published multivariate hypergeometic test to show the TF interactions within the peak.

JunD belongs to the AP-1 family of transcription factors and it is well established that most of the members form homo as well as hetero dimers with other family members [20]. JunD, when forming homo or hetero dimers with the Jun family of proteins, prefers binding to the 7-mer phorbol 12-O-tetradecanoate-13-acetate (TPA)-responsive element (TRE) motif TGA[C/G]TCA; while it binds to 8-mer cAMP-responsive element (CRE) motifs TGACGTCA when it forms hetero dimers with members of the ATF family (Figure 2a) [20]. A ChIP experiment will precipitate the TF as well as the whole TF complex bound to a genomic region. To infer the interaction between two TFs using gene expression data a method has been developed based upon the hypothesis that if two TFs act cooperatively they both should bind to the promoter of their target genes, and the common targets of the two TFs should be more correlated compared to their binding alone (Figure 2b) [16]. We tested this hypothesis in WKY and WKY.L*Crgn2* using LPS ChIP-Seq peaks binding in the promoter region of genes (±500 bp of the TSS) (Figure 2b), and determined whether known, experimentally verified, non-AP-1 interacting partners of JunD (i.e., EP300, YY1, DDIT3 and SMAD3 from BioGrid database) can be inferred using similarity of gene expression profiles [21]. A multivariate hyper-geometric test confirmed that these TFs co-operatively interact in WKY while in WKY.L*Crgn2* the interaction was impaired (Table 1). This supports the usefulness of gene expression data to detect interacting TFs, and suggests that the reduction in JunD’s expression level not only decreases the number of peaks in the congenic but also reduces JunD’s interaction with other TFs.

**Table 1:** Predicting known JunD interacting proteins using gene expression data

**Gene names**	**Pubmed ID**	**Matrix ID**	**WKY LPS ChIP-seq peaks interaction P-value**	**WKY.L*Crgn2 *ChIP-seq peaks interaction P-value**
EP300	16264271	P300_01	2.34e −23	No binding sites observed underneath the peaks
YY1	17510411	YY1_01	0.08	1
		YY1_02	0.002	0.84
DDIT3	10523647	Chop	3.16e −13	0.32
SMAD3	10220381	Smad3	7.9e −12	0.73

### Inferring genome-wide TF-interactions with JunD using ChIP-Seq data

JunD’s co-operative interacting partners for the WKY LPS datasets were predicted on the basis of co-occurrence of binding site motifs within ChIP-Seq peaks using SpaMo [5]. This was investigated in WKY where we observed a higher number of peaks in response to LPS stimulation as compared with WKY.L*Crgn2* (Figure 1B), which had also 20 fold less JunD expression. Where TRE and CRE AP-1 motifs were used as primary motifs, we identified 168 (TRE) and 91 (CRE) secondary transfac motifs respectively, representing 107 and 58 TFs respectively (Additional file 1: Table S1 and Additional file 2: Table S2), including ~50% known interacting TFs and suggesting widespread TF-interactions with JunD.

### Combining gene expression profiling with TF-targets identified by ChIP-Seq analysis

**Figure 3:**
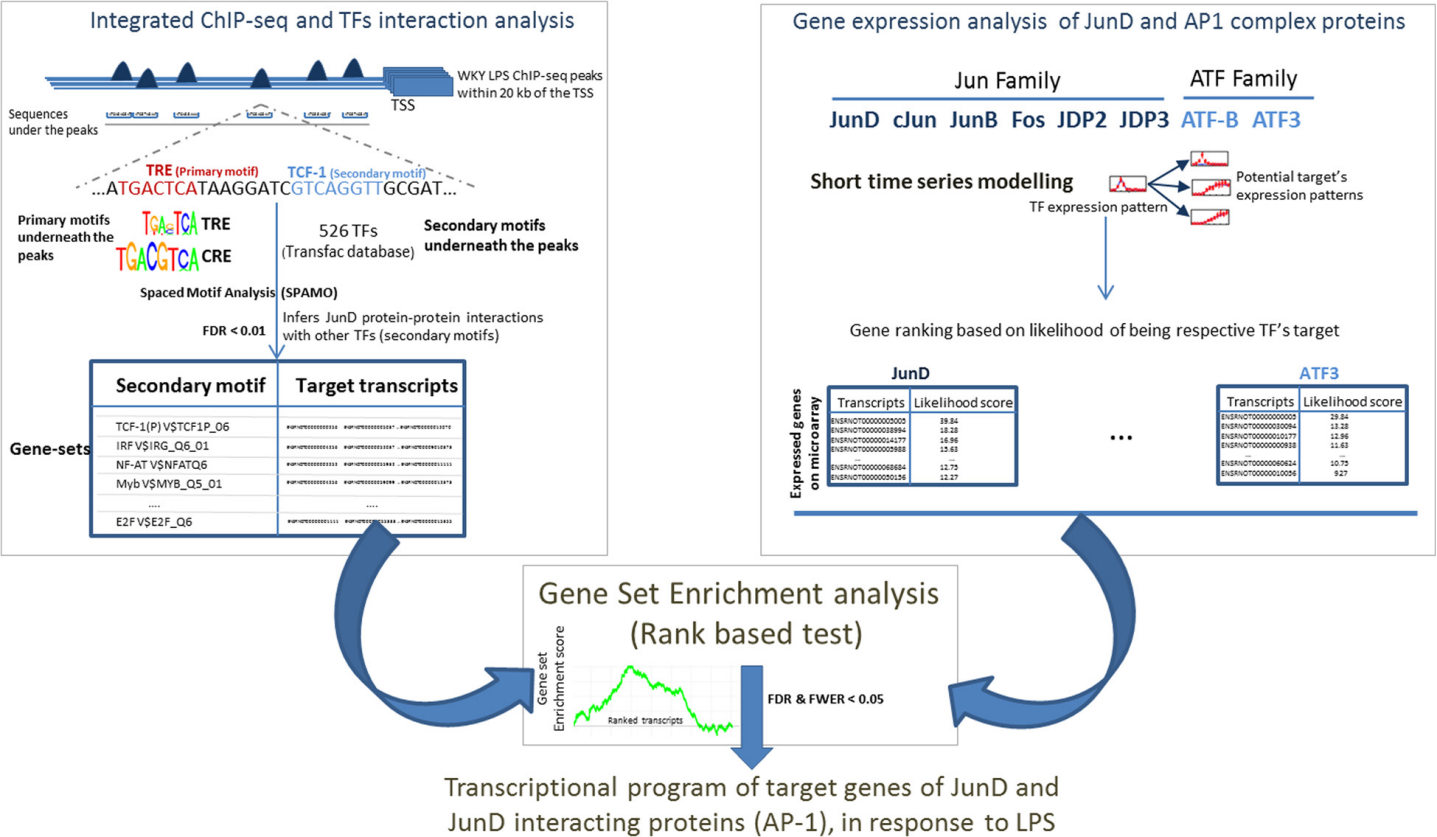
**Integrating ChIP-Seq data with gene expression data.** Methodology for integrating ChIP-Seq data with the time-course microarray gene expression data has been described here. The left hand panel describes the methodology for characterising the co-localised motifs. We have employed Spaced Motif Analysis (SPAMO) for characterising the set of motifs that were observed to occur together (P-value < 0.01). We have performed this analysis considering TRE and CRE as primary motifs. The right hand panel describes the methodology for predicting TF targets based upon gene expression data. We have used a modelling based approach for the short time-series, to rank the microarray genes based upon the likelihood of being targets of AP-1 family members, Jun/ATF. We then performed Gene Set Enrichment analysis to characterise over-representation of the transcripts containing peaks with SPAMO inferred interacting partners.

**Figure 4:**
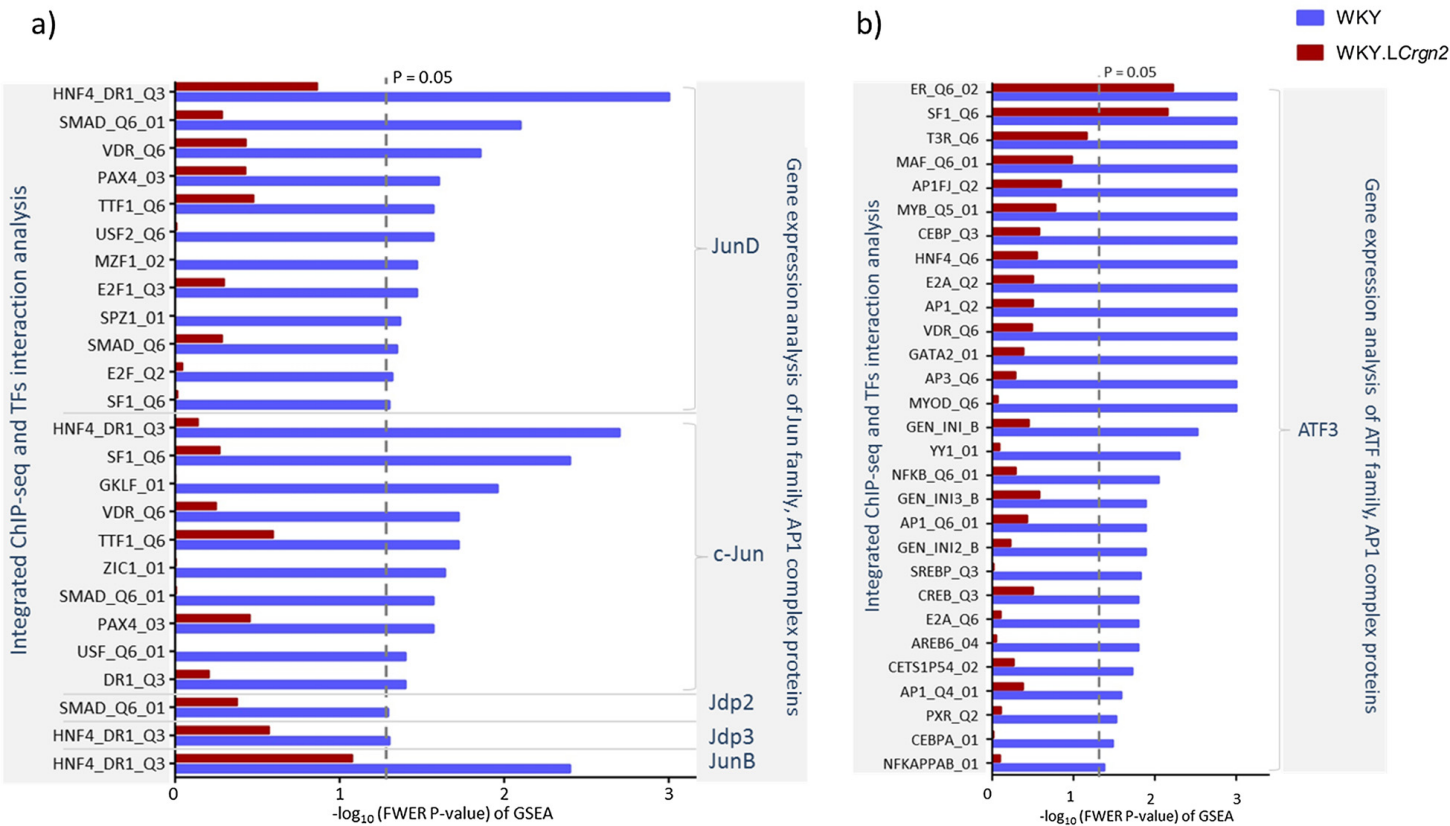
**Gene Set Enrichment analysis for AP-1 complex family members. a)** Summary of the over-represented co-localised TFs with TRE motifs after integration. **b)** Summary of over-represented co-occurring TFs with CRE motifs after integration. For complete list please refer to Additional file 3: Table S3.

We aim to characterise genome-wide direct targets of JunD, and AP-1 complexes that are driven by JunD. To investigate the mRNA activity of the targets of JunD complexes we focused on AP-1 family members that were represented on the microarray: JunD, JunB, c-Jun, JDP2, JDP3, Fos, Fosl1, ATF3 and B-ATF. We used Gene Set Enrichment Analysis (GSEA) [18,19] to test whether the target genes of TF partners interacting with JunD (identified by ChIP-Seq analysis, Additional file 1: Table S1 and Additional file 2: Table S2) are overrepresented amongst the TF-targets that were predicted based on the similarity of expression profiles with AP-1 TFs (Figure 3). Since there is 20 fold less JunD expression in WKY.L*Crgn2,* we considered only WKY LPS for identification of TF-target genes by ChIP-Seq analysis. LPS time course expression data in WKY and WKY.L*Crgn2* were used to predict TF targets based on the TF expression profile (see Methods). TFs interacting with JunD family members that were detected by the GSEA analysis are reported in Figure 4 and Additional file 3: Table S3.

Integrative analysis of ChIP-Seq peaks containing TRE motif targets of the Jun family members and their gene expression resulted in the identification of 16 unique gene sets, corresponding to 13 TFs. These were significantly enriched in WKY, but none of them were enriched in WKY.L*Crgn2* at 5% FWER (Figure 4). Twelve out of the sixteen gene-sets (75%) were specifically associated with JunD and corresponded to ten interacting TFs.

The CRE motif is the preferred motif for ATF homo and hetero dimers (Figure 4b and Additional file 3: Table S3). The integrative analysis of the ATF family of TFs identified only two gene-sets to be significantly enriched in WKY.L*Crgn2* while 32 gene-sets (corresponding to 24 TFs) showed significant enrichment in WKY (FWER < 5%). Potential interaction of the identified TFs with JunD or ATF3 were validated using manual and recently published Protein Interaction information Extraction (PIE) [22] search and have identified that 74% (17 TFs) of the putative JunD-ATF3 interacting partners were previously known to interact with either JunD or ATF3 (Additional file 4: Table S4). Altogether, these results suggest that the interaction of TFs with JunD and the regulation of target genes upon LPS stimulation are affected by JunD genotype as demonstrated by the reduced JunD expression and impaired TF interactions in WKY.L*Crgn2*.

### Transcriptional response to LPS stimulation

We identified genes differentially expressed (DE) due to LPS stimulation by comparing genome-wide expression profiles at each time point with respect to the basal state. At 5% false discovery rate (FDR), 7,510, 11,617 and 9,808 genes were differentially expressed in WKY at 2,4 and 8 hour time points respectively; while a similar number of significantly differentially expressed genes was observed in WKY.L*Crgn2* (Additional file 5: Table S5). We investigated the efficacy of the LPS treatment in rat BMDM by analysing the transcriptional response of a set of established primary and secondary response genes, previously identified in the mouse by Ramirez-Carrozzi et al. [23]. Consistent with the mouse data, we found significant up-regulation of both primary and secondary response genes at initial time-points of the LPS treatment in WKY and WKY.L*Crgn2* strains (Additional file 6: Table S6). To explore how LPS stimulation affects the transcriptional regulation of target genes of JunD and interacting TF partners, for each time point of the LPS time course we performed over-representation analysis of inferred JunD target genes within the DE genes. To distinguish between activation and repression activity of JunD interacting TF partners, the over-representation test was carried out separately for up- and down-regulated target genes (Additional file 1: Table S1). For the predicted targets of the Jun/AP-1 complex (TRE motifs), we found significant enrichment only for the HNF4/DR1 TF, where its target genes were DE at the 4 hr time point (Additional file 7: Table S7). A similar enrichment analysis was performed for the ATF family (CRE motifs), which showed 30 gene-sets (out of 32) as specifically enriched amongst the set of up-regulated genes in WKY (Table 2 and Additional file 8: Table S8). This is consistent with ATF3 binding as a homo-dimer and acting as a repressor of transcription but in a hetero-dimer with JunD it acts as an activator of the transcription [24]. No significant enrichments were observed in WKY.L*Crgn2* for both AP-1 and ATF TFs interacting with JunD. These results suggest that ATF interaction with JunD might be important for macrophage activation in WKY, with the most significant transcriptional changes occurring at 4 hrs post LPS stimulation.

**Table 2:** Over-representation analysis for the genes significantly associated after integration with genes that are differentially regulated during LPS stimulation

		**WKY**						**WKYL.*Crgn2***					
		**Control v 2 hrs**		**Control v 4 hrs**		**Control v 8 hrs**		**Control v 2 hrs**		**Control v 4 hrs**		**Control v 8 hrs**	
**Transfac matrix ID**	**TF**	**Upregulated TF-targets**	**P-value**	**Upregulated TF-targets**	**P-value**	**Upregulated TF-targets**	**P-value**	**Upregulated TF-targets**	**P-value**	**Upregulated TF-targets**	**P-value**	**Upregulated TF-targets**	**P-value**
GATA2_01	GATA2	39	NS	153	3.38e-07	105	0.04	36	NS	102	NS	96	NS
GATA2	GATA2	39	NS	153	3.38e-07	105	0.04	36	NS	102	NS	96	NS
MYOD_Q6	MYOD	31	NS	90	1.94e-06	63	0.01	28	NS	66	0.009	59	0.03
MAF	MAF	51	NS	168	5.62e-06	114	NS	44	NS	105	NS	106	NS
T3R	T3R	47	NS	178	5.82e-06	127	0.05	44	NS	121	NS	120	NS
E2A_Q6	E2A	33	NS	96	7.29e-06	69	0.01	32	NS	69	0.03	61	NS
AP1_Q6_01	AP1	30	NS	118	3.86e-05	89	0.01	28	NS	80	NS	82	0.042
CEBPA_01	CEBPA	19	NS	93	5.35e-05	60	NS	22	NS	61	NS	56	NS
AP3_Q6	AP3	40	NS	146	7.09e-05	101	NS	35	NS	99	NS	94	NS
CREB_Q3	CREB	31	NS	120	0.0001	89	0.03	31	NS	80	NS	82	NS

Top-ten most significantly enriched interacting TF at 4 hrs WKY time-point is listed here, for complete list please refer to Additional file 8: Table S8. NS, P > 0.05.

**Figure 5:**
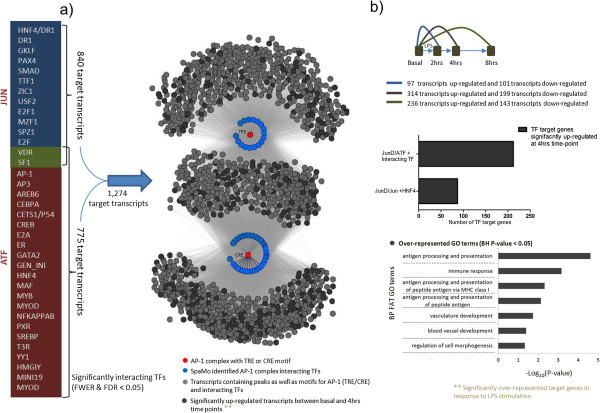
**JunD/AP1 regulatory network in BMDM macrophages. (a)** Integrative analysis identified 36 TFs interacting with AP1 complex (JunD/JUN or JunD/ATF), which regulated expression of 1,274 target genes in LPS-stimulated BMDM macrophages in WKY strain. TF and TF-target genes are represented as nodes (circles) where JunD dimers are highlighted in red, the interacting TF are shown in blue and the TF-target genes in grey. Dark grey colour indicate TF-target genes that are significantly up-regulated upon LPS stimulation at 4 hr. **(b)** Number of differentially expressed genes during LPS-stimulation time course (top); enrichment of up-regulated target genes for JunD/ATF as compared with JunD/Jun TF complex (middle); GO annotation of JunD dimer target genes up-regulated at 4 hr LPS in BMDM (bottom).

Combining all Jun/AP-1 and ATF target genes, we delineated a large set of 1,274 transcripts (Additional file 9: Table S9) that were found to be over-represented in the WKY DE gene dataset (P < 0.05), but not in WKY.L*Crgn2* (Figure 5). This over-representation was due to 314 genes that were up-regulated in WKY at the 4 hr LPS time point, and these were significantly enriched for Gene Ontology terms including immune response (P = 6.89e-04), antigen processing and presentation (P = 2.41e-05) as well as terms linked to morphogenesis (P = 0.047) and vessel development (P = 0.04) (Figure 5). Taken together, these data identify a set of TFs that interact with JunD and their regulated target genes in WKY upon LPS stimulation. This regulatory network centred on JunD, and the interacting TFs is schematically summarised in Figure 5.

## Discussion

Identification of functionally active TF targets in specific cell type can provide insights into crucial biological processes, which might underlie the patho-physiology of disease. A genome-wide TF binding profile can be obtained by ChIP-Seq analysis and, based on the definition of the gene promoter length, ChIP-Seq peaks can be associated with the nearest gene’s TSS [1]. However, association of the TFBS to the nearest gene TSS is not sufficient to establish the downstream gene as a TF target since the TFBS might not be active in a given cell-type or cell-activation status [1]. Hence, the characterization of active TFBS and functionally active TF-target genes in a specific cellular context remains to be elucidated. In this study we aimed to identify functional TFBS by integrating TF targets identified by ChIP-Seq analysis with TF target activity that was predicted using gene expression data. We developed a computational framework for the integrative analysis and implemented it in a rat model for Crgn, where we focus on the JunD TF which has been previously shown to be a primary determinant of macrophage activation [9]. The congenic model (WKY.L*Crgn2*) has been comprehensively tested in previous studies where they have shown that the JunD expression levels are significantly higher in WKY when compared with the congenic [4] and by performing TransAM assay it has been shown that the canonical binding of AP-1 is significantly greater in WKY compared to WKY.L*Crgn2*[9]. The aim of this study is to characterise the direct targets of JunD complexes in WKY and its differential function in WKY.L*Crgn2* during macrophage activation.

After LPS stimulation, due to changes in the JunD expression levels between WKY and WKY.L*Crgn2* the number of JunD ChIP-Seq peaks as well as interactions of AP-1 with other TF was impaired*.* These observations suggest that JunD plays an active role during macrophage activation in WKY while its role is diminished in the congenic strain. The AP-1 family has been implicated in macrophage activation [9,25], and here we were interested in characterising the role of JunD driven AP-1 complexes during macrophage activation. AP-1 complexes can be broadly be classified into Jun and ATF families. JunD is capable of forming homo as well as hetero dimers: when forming hetero dimers with the Jun/Fos family it prefers the TRE motif, while with the ATF family it prefers CRE motifs. However, by sequence analysis of TF binding motifs, it is not possible to sub-classify the members of AP-1 families, since they recognise the same TF binding motifs. So, each member of the Jun/Fos and ATF families was considered separately for investigation of target gene expression using microarrays. We have modelled the gene expression profiles of the TFs and their targets to estimate the likelihood of a transcript to be a target of JunD/AP-1 complexes. Moreover, using spaced motif analysis, JunD interacting partners were predicted, ~50% of which were previously experimentally validated (Figure 3).

Considering the differences in the physiological levels of JunD between WKY and WKY.L*Crgn2*, it can be anticipated that the ChIP-Seq derived TF target data should be more concordant with the gene expression based TF’s targets in WKY compared to WKY.L*Crgn2*. The HNF4/DR1 was the only gene-set that showed an enrichment with the 4 hr up-regulated significantly differentially expressed genes during WKY and not in WKY.L*Crgn2*. Interestingly, the entire Jun family member showed enrichment with HNF4/DR1, suggesting that HNF4 might be important for macrophage activation. HNF4 has been previously linked to chemokine induced inflammatory response [26] and lipid metabolism.

The ATF family also showed an exclusive enrichment for 29 out of 31 gene-sets in WKY, while only 2 gene-sets were observed to be significantly enriched in WKY.L*Crgn2*. This is not unexpected, considering that the congenic has lower expression of JunD. Although the gene-set enrichment was not exclusive to WKY, Fisher’s exact test for 27 gene-sets showed exclusive enrichment with WKY up-regulated genes during macrophage activation at the 4 hour LPS time point. This analysis led to the identification of 866 genes that showed an enrichment of GO terms associated with antigen processing and presentation, signalling cascades and nucleic acid metabolic processes. ATF’s interaction with JunD in the context of macrophage activation has not been studied in depth. A previous study has shown that their interaction regulates the chemokine RANTES (regulated and normal T cell expressed and secreted), which is critical for macrophage activation [27]. This suggests that ATF3 and/or B-ATF could be important for WKY macrophage activation.

ATF3 primarily acts as a transcriptional repressor but when it forms a hetero dimer with JunD it acts as an activator of transcription [24]. ATF3 has been previously hypothesised to be a global regulator of macrophage activation [28]. It has been shown before that ATF3 is up-regulated early after LPS induced toll-like receptor (TLR) engagement which together with NF-κB constitutes a negative feedback mechanism to down regulate TLR [25]. Although Gilchrist et al. observed over-representation of ATF3, AP-1 and NF-κB target genes, they primarily focused on the co-operative interaction of ATF3 with NF-κB. Hence, the role of ATF3 in the context of JunD was not studied in detail. With our integrated analysis however, we have identified direct targets of the ATF3-JunD complex that might be important for macrophage activation.

B-ATF is another member of the ATF family that is induced in T cells and natural killer cells upon stimulation and is a proposed to be a therapeutic target for immunotherapies. It has also been shown to be critical for the T_H_17 cell inflammatory immune response [29], contrary to the belief that B-ATF is a suppressor of AP-1 gene expression [30].

The ATF-JunD hetero-dimer was observed to be associated with 22 TFs and their targets were significantly enriched in the 4 hr LPS time-point comparison with the basal state while targets for 7 TFs were observed to be enriched in the 8 hrs time-point comparison. This suggests that hetero-dimer interaction of JunD with ATF family members might be more important during macrophage activation than the hetero-dimers formed with the Jun family.

## Conclusions

In this study we have developed a methodology not only to predict direct targets for TFs but also to infer functional co-operative interactions. In this study we have only considered the AP-1 complex, but this can be applied to any TF complex. By combining gene expression data with ChIP-Seq profiles we have identified 1,274 genes which are directly associated with JunD and have shown their involvement during macrophage activation. Considering that *JunD* is a gene with major differential expression between WKY and WKY.L*Crgn2*, these 1,274 genes are likely to be direct targets of *JunD.* Our data suggests interplay of JunD with the ATF family and HNF4 during macrophage activation, which was previously unappreciated. Further study of the interplay between these two TFs will provide the basis for understanding *JunD-*mediated macrophage activation, enabling identification of novel targets for modulating macrophage function.

## Competing interests

The authors declare that they have no competing interests.

## Author’s contributions

PKS developed and implemented the methodology with contributions from EP. RPH and JB carried out the laboratory experiments. PKS and EP wrote the manuscript with contributions from TJA, RH and JB. All authors read and approved of the final manuscript.

## Supplementary Material

Additional file 1: Table S1 SpaMo inferred protein-protein interaction using TRE motif: Spaced motif analysis resulted in 170 transfac TF matrices were predicted to be significantly enriched with TRE 7-mer AP-1 motif. A TF can have multiple binding sites which is represented by multiple Transfac matrix IDs. 170 Transfac IDs mapped to 108 unique TF out of which 54 were known interacting partners of AP-1 complex. Click here for file

Additional file 2: Table S2 SpaMo inferred protein-protein interaction using CRE motif: Spaced motif analysis resulted in 92 transfac TF matrices were predicted to be significantly enriched with CRE 8-mer AP-1 motif. 92 Transfac IDs mapped to 66 unique TF out of which 36 (54%) were known interacting partners of AP-1 complex. Click here for file

Additional file 3: Table S3 Gene expression integration results obatined with CRE motif. This table shows top ChIP-seq peaks with the over-represented motifs. For each TF we provide the number of target transcripts for the TF complex and enrichment results for both WKY and congenic (NES: Normalised enrichment score, FWER p-value: Family wise error rate p-value). We observed that with an exception of two TFs ER and SF1 rest of the TFs were exclusive to WKY. Click here for file

Additional file 4: Table S4 This table summarises the results obtained by performing literature search for experimentally validated protein-protein interactions using Protein Interaction information Extraction (PIE) search (Kim et al., 2012) followed by manual curation of these data. Click here for file

Additional file 5: Table S5 List of differentially expressed transcripts. This table contains list of differentially expressed transcripts in WKY and its congenic when compared with the basal time point. Expression fold change with respect to the basal state are given for each gene and condition. Click here for file

Additional file 6: Table S6 Gene expression changes in primary and secondary response genes following LPS stimulation in WKY and WKY.L*Crgn2* BMDMs. This table reports the fold-changes of significantly differentially expressed genes (FDR < 5%) that matched previously identified primary and secondary response genes identified in mouse BMDMs by Ramirez-Carrozzi et al. The set of genes highlighted in grey are the genes that were either not differentially expressed or had very expression values or didn’t had mouse orthologous genes in rats. Click here for file

Additional file 7: Table S7 Over-representation analysis of the genes obtained after ChIP-seq gene expression integration with the TRE motif. This table summarises the results obtained after performing Fisher’s exact test for gene-sets obtained by considering TRE motifs against the significantly differentially up-regulated set of transcripts in WKY and its congenic. NS: Not significant at P-value < 0.05. Click here for file

Additional file 8: Table S8 Over-representation analysis of the genes obtained after ChIP-seq gene expression integration with CRE motif. This table summarises the results obtained after performing Fisher’s exact test for gene-sets obtained by considering CRE motifs against the significantly differentially up-regulated set of transcripts in WKY and its congenic. NS: Not significant at P-value < 0.05. Click here for file

Additional file 9: Table S9 List of transcripts obtained after integration analysis. 1,274 transcripts obatined after integration of the gene expression with the ChIP-seq profile are listed here. Click here for file
