# Supplementary material for: JunD/AP1 regulatory network analysis during macrophage activation in a rat model of crescentic glomerulonephritis
Source: BMC Syst Biol. 2013 Sep 22;7:93. doi: 10.1186/1752-0509-7-93 (PMC3849178; doi:10.1186/1752-0509-7-93)
Supplement: Additional file 4: Table S4 — This table summarises the results obtained by performing literature search for experimentally validated protein-protein interactions using Protein Interaction information Extraction (PIE) search (Kim et al., 2012) followed by manual curation of these data. [file 1752-0509-7-93-S4.docx]

**Suppl. Table-4:** This table summarises the results obtained by performing literature search for experimentally validated protein-protein interactions using Protein Interaction information Extraction (PIE) search ([Kim et al, 2012](#_ENREF_3)) followed by manual curation of these data.

| **Transcription Factors** | **Official Gene Names** | **PubmedID** | **Experiment** |
| --- | --- | --- | --- |
| AP1 | Jun/fos | 2501150 | affinity chromatography |
| AP3 | AP3 | 11961136 | Preparative electrophoretic mobility shift assays coupled to Western analysis |
| ETS1 | ETS1 | 7648395 | Co-immunoprecipitation |
| General initiation factor | TFIID/TBP/ | 7848298 | affinity chromatography |
| YY1 | YY1 | 8753872 | EMSA |
| MAF | MAF | 8264639 | EMSA |
| GATA2 | GATA2 | 7623817 | Co-immunoprecipitation |
| MYB | MYB | 9566892 | Co-immunoprecipitation |
| SF1 | SF1 | 9753627 | co-transfection of expression vectors in conjunction with mutation analysis |
| ER | ESR1 / ESR2 | 10500157 | EMSA |
| VDR | VDR | 10330159 | EMSA |
| Myod | MYOD | 7769693 | co-transfection of expresison vectors inconjuction with mutation analysis |
| CREB | CREB1 | 16373341 | EMSA |
| HNF4 | HNF4a | 16729332 | co-immunoprecipitation |
| cebpa | CEBPa | 17082780 | co-immunoprecipitation |
| CEBP | CEBPa / CEBPb | 18026136 | co-immunoprecipitation |
| NFKB | v-REL | 7520524 | DNA transfection and CAT analysis |
| ZEB1 | ZEB1 | N/A | N/A |
| HMG | HMGa1 | N/A | N/A |
| SREBP | SREBP | N/A | N/A |
| E2A | TCF3 | N/A | N/A |
| PXR | NR1i2 | N/A | N/A |
| Thrb | THRB | N/A | N/A |
| Muscle V$MINI19 B | - | N/A | N/A |
